# To Build a Bridge for International Academic Exchange of Critical Care Medicine

**DOI:** 10.1007/s44231-022-00017-0

**Published:** 2022-10-28

**Authors:** Chengzeng Wang

**Affiliations:** The First Affiliated Hospital of Zhengzhou University, Zhengzhou, China

On the first anniversary of the publication of *Intensive Care Research*, on behalf of the First Affiliated Hospital of Zhengzhou University, I would like to extend my warm congratulations to the editorial board of *Intensive Care Research* and my heartfelt thanks to the readers, authors and critical care experts who support the journal.

As the first “Diamond Open Access” English journal of critical care medicine in China, *Intensive Care Research* supervised by Zhengzhou University and published by Springer Nature Press is co-sponsored by the First Affiliated Hospital of Zhengzhou University and the Chinese Research Hospital Association. Professor Tongwen Sun is the editor-in-chief of the journal. Since its inception, the journal has published 6 issues through the unremitting efforts of the editorial board members and provided an updated and convenient academic exchange platform for critical care scholars, attracting the attention of domestic and foreign critical care medical experts.

The First Affiliated Hospital of Zhengzhou University, founded in September 1928, has a history of nearly 100 years. It has entered a new stage of high-quality development, and has formed a new pattern of collectivization development of “one hospital in four districts”. The hospital continues to adhere to and strengthen the overall leadership of the Party, innovate in the new system, new trend, new efficiency, new energy and new culture, promote the expansion and balanced distribution of high-quality medical resources step by step, focus on building a modern hospital management system, and actively contribute to the development of Zhengzhou as a National Central City and the construction of Healthy Henan and Healthy China. The four hospitals altogether have 14,341 employees, including 624 professors, 1551 associate professors, 2063 PhD or MD degree holders, 1 full-time academician, and 17 distinguished academicians. The hospital has 14 intermediate hospitals, 120 clinical and technical departments, 279 wards, 10,500 beds. With more than 8.28 million outpatient visits per year, more than 680,000 discharged patients per year, and more than 360,000 operations per year, the hospital is leading in the diagnosis and treatment capabilities in China, ranking the 19th in China’s Hospital Ranking (Fudan University) and the 21st in China’s Hospital Ranking by Science and Technology Evaluation Metrics.

Critical care medicine in China started in the 1980s, which was later than that in western countries. Over the past 10 years, the exchange of critical care medicine between China and the world has become increasingly close, with increasing depth and breadth. A large number of Chinese intensivists went abroad and acquired new theories, techniques and methods of critical care medicine through a variety of academic conferences and visits in international medical and research centers, narrowing the gap with the international advanced level. The voices of Chinese critical care medicine are increasingly appearing on the stage of important international academic exchanges. The basic and clinical research is increasingly appearing in top academic journals related to critical care. Top teams in Europe and the United States, as well as international experts, are increasingly paying attention to and participating in Chinese critical care. Large-scale clinical research in China provides us with massive clinical data and unparalleled advantages for clinical research and plays a positive role in promoting the development of critical care medicine in the world. The two-way exchange and integration of Chinese critical care into the international community has greatly promoted the development of Chinese international cooperation in critical care medicine.

In recent years, as a national key clinical specialty, critical care medicine in China has achieved sound and rapid development. Especially since the outbreak of COVID-19, critical care medicine has always been on the front line of fighting against the epidemic, making an extraordinary contribution to the prevention and treatment of the epidemic and protecting people's health. Our hospital has established a complete critical care medical treatment system covering comprehensive ICU, EICU, NICU, RICU, PICU, SICU, CCU, neurological ICU, anesthesia ICU, etc. Its emergency and critical care treatment capacity has topped the country. In the past 5 years, our hospital has published 114 papers in critical care medicine included by Science Citation Index, among which there are more than 50 in the second district (ranking the top 6 ~ 20%) and nearly 10 in the first district (ranking the top 5%). During the COVID-19 epidemic in 2020, Professor Tongwen Sun firstly took the lead in formulating “Chinese Expert Consensus on Diagnosis and Treatment of Severe and Critical Coronavirus Disease 2019” published in both Chinese and English, which greatly reduced the domestic mortality of severe COVID-19. The consensus transmitted China’s experience to the world and played a guiding role in the treatment of severe COVID-19 in countries around the world. The founding of *Intensive Care Research* is an important milestone in the development of the department of critical care medicine in our hospital and also provides an important platform for the domestic intensive care medicine to communicate with its counterpart abroad. It is hoped that the journal can build a bridge for international exchange of Chinese and foreign intensive care medicine, transmitting the voice of China and at the same time drawing on the advanced achievements of the world to benefit Chinese critical care medicine development.

“High is the tide and the broad are the banks. Strong is wind and the sail is full-blown.” The First Affiliated Hospital of Zhengzhou University fully supports the development of *Intensive Care Research*, hoping that the journal will continue to strive hard toward the international first-class journal. It will surely contribute to the “double first-class” construction of Zhengzhou University and rejuvenating the country through science and technology.

